# The Feather Moss *Hylocomium splendens* Affects the Transcriptional Profile of a Symbiotic Cyanobacterium in Relation to Acquisition and Turnover of Key Nutrients

**DOI:** 10.1007/s00248-024-02363-6

**Published:** 2024-03-01

**Authors:** Danillo Oliveira Alvarenga, Anders Priemé, Kathrin Rousk

**Affiliations:** 1 Department of Biology, University of Copenhagen, Universitetsparken 15, 2100 Copenhagen, Denmark; 2 Center for Volatile Interactions, University of Copenhagen, Universitetsparken 15, 2100 Copenhagen, Denmark

**Keywords:** Boreal forests, Nitrogen fixation, Plant–microbe interactions, Sulfur, Symbiosis, Metatranscriptomics

## Abstract

Moss-cyanobacteria symbioses were proposed to be based on nutrient exchange, with hosts providing C and S while bacteria provide N, but we still lack understanding of the underlying molecular mechanisms of their interactions. We investigated how contact between the ubiquitous moss *Hylocomium splendens* and its cyanobiont affects nutrient-related gene expression of both partners. We isolated a cyanobacterium from *H. splendens* and co-incubated it with washed *H. splendens* shoots. Cyanobacterium and moss were also incubated separately. After 1 week, we performed acetylene reduction assays to estimate N_2_ fixation and RNAseq to evaluate metatranscriptomes. Genes related to N_2_ fixation and the biosynthesis of several amino acids were up-regulated in the cyanobiont when hosted by the moss. However, S-uptake and the biosynthesis of the S-containing amino acids methionine and cysteine were down-regulated in the cyanobiont while the degradation of selenocysteine was up-regulated. In contrast, the number of differentially expressed genes in the moss was much lower, and almost no transcripts related to nutrient metabolism were affected. It is possible that, at least during the early stage of this symbiosis, the cyanobiont receives few if any nutrients from the host in return for N, suggesting that moss–cyanobacteria symbioses encompass relationships that are more plastic than a constant mutualist flow of nutrients.

**Supplementary Information:**

The online version contains supplementary material available at 10.1007/s00248-024-02363-6.

## Introduction

Mosses are non-vascular plants that are among the dominant plant groups in many northern terrestrial ecosystems. These bryophytes host a diverse set of symbiotic microbes, such as cyanobacteria, that contribute significantly to both the carbon (C) and nitrogen (N) cycles [1]. Cyanobacteria associated with mosses have been repeatedly found to constitute an important source of new N into northern environments that are abundantly colonized by bryophytes, like boreal forests [2–4].

The amount of N fixed in moss–cyanobacteria associations possibly depends on the host plant. Mosses influence the composition and the N_2_-fixing activities of associated cyanobacterial communities, which show high degrees of host specificity [5–7]. This is at least partly a result of the varying rates in which different moss taxa are colonized by cyanobacteria, a consequence of a combination of biotic factors that include taxon-dependent morphological traits in the hosts [8].

Symbiosis, defined as different species living together in intimate, physical contact [9], comprises a dynamic continuum of interactions ranging between mutualism, commensalism, and parasitism, among which symbionts may transition depending on environmental and ecological conditions [10]. As N_2_ fixation is a very costly process, cyanobacteria-plant symbioses often encompass mutually beneficial relationships where the host reciprocates the increased N_2_-fixing activities of their cyanobionts by supporting them with C [11, 12]. As such, some works have shown that host plants may up-regulate genes involved in photosynthesis [13] and ammonium transport [14] when in contact with cyanobionts. Likewise, symbioses between cyanobacteria and the feather moss *Pleurozium schreberi* and the peat moss *Sphagnum angustifolium* have been proposed to be mutualistic and based on nutrient exchange, as the hosts could potentially transfer C and S to their cyanobionts and receive N in return [15, 16].

It is hard to say if this is a general pattern in cyanobacterial symbioses with mosses because these relationships are much looser than the endophytic relationships cyanobacteria establish with tracheophytes or other bryophytes such as hornworts and some liverworts [11]. Additionally, N_2_-fixing communities in different mosses can be more complex than commonly thought and may also include bacteria other than cyanobacteria [16]. Furthermore, in some bryophyte-cyanobacteria symbioses, increased N_2_ fixation by cyanobionts can be verified within 1 week of contact, but declines sharply after 2 weeks [17]. Thus, the interactions taking place in these symbioses are also likely to fluctuate with time.

Although the importance of moss–cyanobacteria symbioses for some pristine ecosystems cannot be overstated, the molecular mechanisms involved in their establishment and maintenance are still mostly unknown. The few studies investigating gene expression in these interactions suggested that 3–5 weeks of physical contact between moss and cyanobionts results in the upregulation of cyanobacterial genes related to phosphorus (P) uptake, oxidative stress, and N_2_ fixation, while genes related to host defense are downregulated [16, 18]. However, cyanobacterial interactions with other moss species, like the widespread boreal forest feather moss *Hylocomium splendens*, are still unexplored, and it is possible that the characteristics of moss–cyanobacteria interactions change with time, environmental conditions, and/or hosts. Nonetheless, we have recently shown that 1 week of contact between cyanobacteria and *H*. *splendens* shoots or its extracts increases the heterocyte frequency and, consequently, N_2_ fixation rates of the cyanobacteria without significant changes in biomass [19]. Hence, mosses likely influence the activity and morphophysiology of their cyanobionts within days of initiating contact.

Following this previous work, we investigated how a week of close physical contact between *H. splendens* and its N_2_-fixing cyanobiont affects gene expression in both parts. Based on potential nutrient exchange interactions suggested earlier where mosses could provide C and S and receive N from the cyanobiont, we hypothesized that (1) genes linked to N_2_ fixation would be up-regulated in the cyanobiont when in contact with *H. splendens* and genes related to N uptake would be up-regulated in *H. splendens* under symbiosis; (2) genes related to S uptake would be up-regulated in the cyanobiont when co-incubated with *H. splendens*; (3) photosynthesis would be down-regulated in the cyanobiont when in contact with its host; and (4) photosynthesis would be up-regulated in *H. splendens* during the symbiosis with its cyanobiont. Since no reference genomes are available for these organisms and the samples were non-axenic, we used a de novo metatranscriptomic strategy that allowed us to estimate how gene expression in the mosses and the cyanobacteria was affected by their symbiotic relationship.

## Methods

### Moss Sampling and Cyanobacterial Isolation

Shoots of the feather moss *Hylocomium splendens* (Hedw.) Schimp. were collected on August 2020 in boreal forests from northern Sweden located in the larger Arvidsjaur area (64°58′50.0″–66°1′13.6″N, 19°33′57.2″–19°51′8.7″E). At least 20 shoots were collected by hand from different spots (approximately 10) within a 10-m^2^ plot (in two different forests) and merged into a composite sample representing each forest site. Samples were stored at 4 °C in the dark until they were processed. The isolation of a cyanobacterial symbiont was initiated by submerging the shoots into a 250-mL Erlenmeyer flask containing 100 mL of liquid BG-11_0_ medium [20, 21] supplemented with 100 µg·mL^−1^ cycloheximide. After 3 months of incubation at 20 ± 1 °C under a photoperiod of 12 h of light at 40 ± 5 µmol photons·m^−2^·s^−1^ and 12 h of darkness, a *Nostoc*-like cyanobacterial strain capable of N_2_ fixation (named UCTE CB012) was obtained by serially transferring a visible colony to fresh culture medium. The isolation process was followed by frequent microscopy-assisted diagnoses to ensure that a unicyanobacterial culture was obtained.

### Culturing and Moss–Cyanobacteria Co-incubation

Using tweezers, moss shoots were manually separated from soil and debris and transferred to 50-mL tubes containing 25 mL of ddH_2_O, where they were sonicated for 10 min. To reduce the abundance of the preexisting microbial communities in the samples, the shoots were further washed by vortexing them with a sequence of sterile solutions (0.1% Tween 20, 1% NaOCl, and 0.1% H_2_O_2_, each intercalated by ddH_2_O). Next, the shoots were cultured at 12 h of light at 125 ± 5 µmol photons·m^−2^·s^−1^ in 100 mL of BCD medium [22] containing 25 mg·mL^−1^ nystatin, 10 mg·mL^−1^ ampicillin, and 10 mg·mL^−1^ streptomycin. To inspect for residual cyanobacteria, the moss samples were observed with the Olympus BX61 fluorescence microscope using the green excitation filter (Olympus, Tokyo, Japan). Unicyanobacterial cultures of the strain UCTE CB012 were also prepared and incubated under the previously mentioned conditions.

After 3 weeks, the cyanobacterial cultures were concentrated by centrifugation at 4000 × *g* for 10 min and the cells were resuspended in 30 mL of sterile ddH_2_O. Afterward, the moss shoots were once again centrifuged and washed with sterile ddH_2_O. Co-incubations were prepared with four replicates according to three treatments: moss shoots alone (solitary state), cyanobacteria and mosses together (co-incubated state), and cyanobacterial cells alone (solitary state). Cyanobacterial cell solutions were homogenized before each inoculation.

For metatranscriptomic analyses, five *H. splendens* shoots were placed in sterile 50-mL tubes with cotton stoppers and inoculated with 1 mL of the cyanobacterial cell suspension. In parallel with this, three shoots were placed in 20-mL sterile glass vials, which were inoculated with 1 mL of the cyanobacterial cell suspension and sealed with parafilm for N_2_ fixation analyses using the acetylene reduction assay. Treatments containing either mosses or cyanobacteria in isolation were processed in the same manner, but with the organisms being kept in separate containers. Treatments consisting of moss shoots alone received 1 mL of sterile ddH_2_O instead of the cyanobacterial inoculum. The samples were incubated under the same conditions as the culturing of the moss shoots described above.

### Acetylene Reduction Assay

The N_2_ fixation rates of the cyanobacteria when solitary and co-incubated with the moss were estimated based on the acetylene reduction assay (ARA) [23]. After 1 week of incubation, the 20-mL vials prepared in the previous step were sealed with rubber caps and 10% of the head space was replaced with acetylene gas. The vials were returned to the growth chamber for 18 h under the previous conditions. The amount of ethylene produced in the samples was quantified via gas chromatography with the Agilent 8890 GC System equipped with the J&W CarboBOND column (Agilent Technologies, Santa Clara, USA). A standard curve was produced using vials containing different concentrations of 300 ppm ethylene. The amount of ethylene in vials containing washed moss shoots alone was subtracted from the values in the co-incubated samples. An empty, sealed vial incubated with acetylene gas only was also analyzed to subtract any existing concentration of ethylene in the acetylene gas used for the incubation.

To verify whether co-incubation with the feather moss host influences N_2_ fixation rates by its cyanobacterial symbiont, the ARA results obtained were statistically analyzed with R 4.2.2 (https://www.R-project.org/) under the RStudio IDE 2022.12.0 (http://www.rstudio.com/). Normality of the data was checked using QQ plots and Shapiro’s test (*p* = 0.39). Differences in acetylene reduction between solitary cyanobacteria and cyanobacteria co-incubated with moss shoots was evaluated using a one-way ANOVA [24] and Tukey’s honest significant distance [25] tests. The results were visualized with the R package ggplot2 3.4.0 [26].

### RNA Isolation

After 1 week, when a significant increase in co-incubated N_2_ fixation rates was verified (approximately 3 times higher), the cotton stoppers were replaced with the original plastic caps, and the samples were flash-frozen by immersion into liquid N_2_. The tubes were kept at − 80 °C until they were freeze-dried at − 55 °C for 24 h. The dried samples were moved to RNeasy PowerBead Tubes (Qiagen, Venlo, Netherlands), which had their ceramic beads replaced with 150–212-μm acid-washed glass beads (Sigma-Aldrich, Saint Louis, USA). RNA was isolated using the RNeasy PowerPlant Kit (Qiagen) using the protocol provided by the manufacturer. DNA co-isolated from the samples was removed with the RNase-Free DNase Set kit (Qiagen). RNA in the samples was quantified with the Qubit 2.0 fluorometer (Life Technologies, Carlsbad, USA) and RNA integrity was checked with Agilent TapeStation 4200 (Agilent Technologies).

### Library Preparation and Sequencing

rRNA depletion was performed with NEBNext rRNA Depletion Kit (Bacteria) (New England Biolabs, Ipswich, USA) for samples that received cyanobacterial inocula and QIAseq FastSelect–rRNA Plant (QIAGEN) for samples that contained moss shoots. A library was prepared with NEBNext Ultra II RNA Library Prep Kit for Illumina (New England Biolabs) following the recommendations of the manufacturer. Briefly, enriched RNAs were fragmented according to manufacturer’s instruction, and first- and second-strand cDNA were subsequently synthesized after cDNA fragments were end-repaired and adenylated at 3′ ends. A universal adapter was ligated to cDNA fragments followed by index addition and library enrichment with limited cycle PCR.

The sequencing libraries were validated on Agilent TapeStation 4200 and quantified with Qubit 2.0 as well as by quantitative PCR (KAPA Biosystems, Wilmington, USA). The sequencing libraries were clustered on a flowcell lane. After clustering, the flowcell was loaded on a NovaSeq 6000 (Illumina, San Diego, USA) according to manufacturer’s instructions. The samples were sequenced using a 2 × 150-bp paired end configuration. Image analysis and base calling were conducted by the NovaSeq Control Software 1.8 (Illumina). Raw sequence data (.bcl files) generated were converted into fastq files and de-multiplexed using bcl2fastq 2.17 (Illumina) with one mismatch allowed for index sequence identification. Both library preparation and sequencing were performed by Genewiz (Azenta Life Sciences, Chelmsford, USA). The RNAseq data obtained in this study was deposited in the NCBI GEO database under the accession numbers GSM7688832–GSM7688843.

### Metatranscriptomic Analyses

The raw sequences were quality-trimmed and adapters were removed with Trimmomatic 0.36 [27] and a de novo assembly was performed with Trinity 2.14.0 [28] using the default parameters. Annotation and mapping were performed with the Comparative Metatranscriptomics Workflow 1.0.0 software [29] Non-coding RNA was filtered from the assembly and the trimmed reads were mapped back to the remaining contigs with BWA 0.7.17-r1188 [30]. Transcripts with expression lower than 1 were removed. The quantified contigs were mapped to the MD5nr database [31] using SWORD 1.0.4 [32] and functional hierarchies were annotated based on the eggNOG database [33].

To eliminate potential contaminants (i.e., sequences from chloroplasts, mitochondria, and those not affiliated with cyanobacteria or mosses) in the assembled contigs, Kraken 2.1.2 with the PlusPFP 9/19/2020 database [34] was used for identifying and retrieving sequences that belonged to classes Cyanobacteria and Bryopsida. Genes with less than 10 mapped reads were discarded from the dataset and the selected contigs were used for evaluating differential gene expression (DGE) between the solitary (moss or cyanobacteria) and the co-incubated samples. The DGE analysis was performed using R 4.2.2 with the package DESeq2 3.16 [35].

## Results

Trimming and quality filtering resulted in an average of 6,516,423 high-quality read pairs (79% of the sequenced read pairs) for treatments containing moss and cyanobacterial samples in their solitary states. Samples having both moss and cyanobacteria in the co-incubated state treatment had an average of 11,899,329 read pairs remaining after trimming, or 76% of the original dataset. The de novo assembly resulted in an E85N50 of 2178 bp. Alignment rates of reads to the assembled transcriptomes were above 83% in all sequenced samples.

**Fig. 1:**
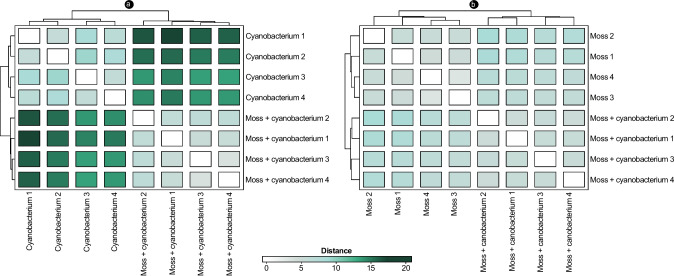
Heatmap representations of distance matrices of variance-stabilizing transformed transcriptomic data from the cyanobacterial strain UCTE CB012 (**a**) and the feather moss *H. splendens* (**b**) in the co-incubated and solitary states. Cyanobacterium: strain UCTE CB012 incubated by itself. Moss: *H. splendens* incubated by itself. Moss + cyanobacterium: co-incubation of *H. splendens* and UCTE CB012. *n* = 4

**Fig. 2:**
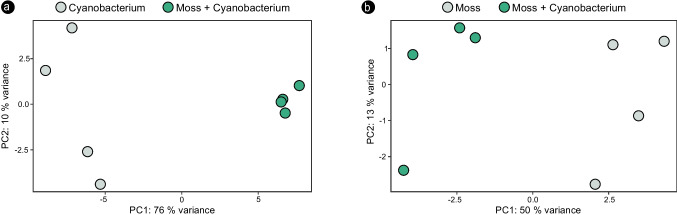
Principal component analyses comparing variance-stabilizing transformed transcriptomic datasets of the cyanobacterial strain UCTE CB012 (**a**) and the feather moss *H. splendens* (**b**) in the solitary and co-incubated states. Cyanobacterium: strain UCTE CB012 incubated by itself. Moss: *H. splendens* incubated by itself. Moss + cyanobacterium: co-incubation of *H. splendens* and UCTE CB012. *n* = 4

Contact with the feather moss led to a substantial change in the gene expression of the cyanobiont UCTE CB012 when compared to when it was incubated alone (Fig. 1a). A principal component analysis showed that 76% of the variance in the cyanobacterial transcriptome could be explained by the differences in gene expression between samples in the co-incubated and solitary states (Fig. 2a). *H. splendens* also showed significant differences in gene expression, but at a lower degree than its cyanobiont, as the differences between the co-incubated and solitary states of the host were estimated to explain 50% of the variance in the *H. splendens* transcriptomes (Figs. 1b and 2b).

**Fig. 3:**
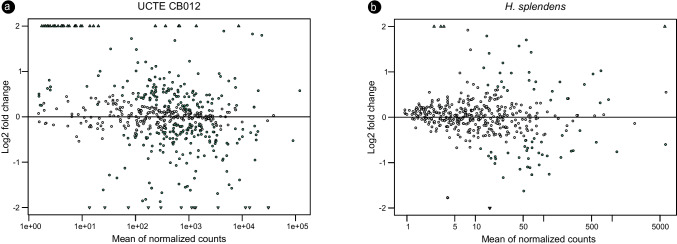
MA plots comparing the log2 fold changes in differential gene expression between the co-incubated and solitary states of the cyanobacterium UCTE CB012 (**a**) and *H. splendens* (**b**) after 1 week. Genes that were significantly up-regulated (positive values) or down-regulated (negative values) in the co-incubated states (*p* < 0.05) are highlighted in green. Triangular spots represent data that extrapolates the limits of the *y* axis. *n* = 4

Gene expression in the cyanobiont was more impacted by contact with the feather moss, with 305 genes presenting significant differential expression (*p* < 0.05) in the cyanobacterial transcriptome in contrast with 54 of the host’s transcripts that were significantly affected by this interaction (Fig. 3). In the UCTE CB012 transcriptome, log2 fold changes (LGF) varied from − 0.158 (in a gene predicted to encode a sugar transferase) to 8.187 (nucleoside diphosphate kinase), while *H. splendens* presented LGFs varying from − 0.583 (GTPase SAR1) to 4.08 (an unknown protein).

**Fig. 4:**
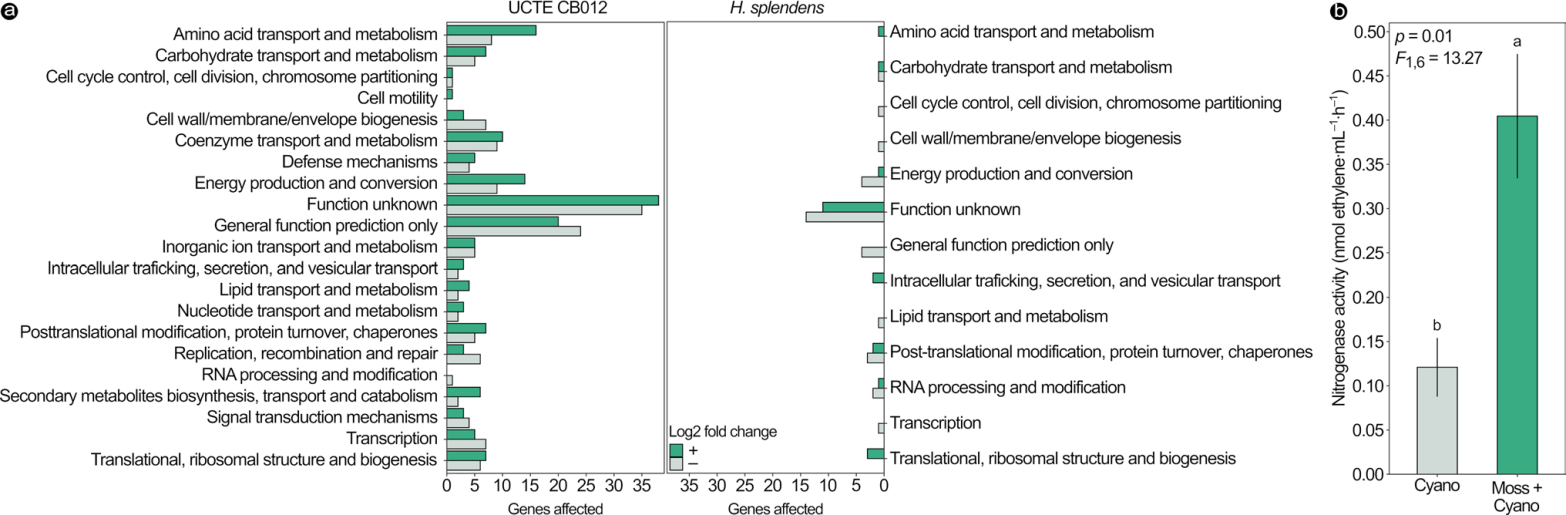
Changes in gene expression and N_2_ fixation activity in the symbiosis between the cyanobacterium UCTE CB012 and the feather moss *H. splendens*. **a** Number of genes in functional categories from the Cluster of Orthologous Genes database that presented significant positive or negative differential expression under co-culture between the cyanobiont UCTE CB012 and *H. splendens*. **b** Increased N_2_ fixation activity in colonies of the strain UCTE CB012 in co-incubation with shoots of *H. splendens* as revealed by acetylene reduction assays. Bars represent average values and lines indicate standard errors, while letters above bars in b represent significant differences observed with the Tukey test (*p* < 0.05). Cyano: strain UCTE CB012 incubated by itself. Moss + cyano: co-incubation of *H. splendens* and UCTE CB012. *n* = 4

Most of the genes that had significant differential expression under co-incubation in both the cyanobacterium and the feather moss transcriptomes are currently classified under unknown functional categories or have general function prediction only (Fig. 4a). Nevertheless, UCTE CB012 also had a large number of genes knowingly related to amino acid transport and metabolism being differentially expressed during its physical contact with its host (Fig. 4a). These changes were reflected in acetylene reduction assays, as colonies of UCTE CB012 incubated under the same conditions indeed increased their N_2_ fixation rates after a week of contact with *H. splendens* shoots, presenting an average value that was approximately 3 times the activity of the colonies that were incubated alone (*F*_1,6_ = 14.39, *p* = 0.01) (Fig. 4b).

In the UCTE CB012 transcriptome, genes encoding proteins involved with N_2_ fixation (including cyanophycinase and the nitrogenase subunit NifH) and the biosynthesis of various amino acids were indeed up-regulated in the co-incubated state (Table 1; Supporting Information Table S1). Nevertheless, the degradation of selenocysteine and threonine was up-regulated, while genes involved with the biosynthesis of other amino acids (methionine, cysteine, threonine, leucine) were down-regulated in the cyanobiont. ABC transporters involved with sulfur uptake were also down-regulated in the cyanobiont transcriptome (Table 1). Two copies of genes encoding the electron carrier ferredoxin were up-regulated, while one copy was down-regulated (Table 1). Most genes related exclusively to photosynthesis did not present significant differential expression in the cyanobacterium in its co-incubated state. The exceptions were genes encoding PsaA and PsaB bind P700, which were significantly up-regulated (LGF 0.601, *p* < 0.001), and the photosystem II D2 protein M, which was down-regulated (LGF − 0.207, *p* = 0.005) (Table 1; Supporting Information Table S1).

**Table 1:** Differential expression of genes involved with nutrient metabolism in the cyanobacterial strain UCTE CB012 after a week of co-incubation with the feather moss *H. splendens*

Gene code		Log2 fold change	*p*	Metabolism	Function	Possible role in symbiosis
Up-regulated	COG2876	1.862	< 0.001	Amino acid transport and metabolism	3-Deoxy-D-arabino-heptulosonate 7-phosphate synthase	Biosynthesis of tryptophan|phenylalanine|tyrosine
	COG0160	0.572	0.001	Amino acid transport and metabolism	4-aminobutyrate aminotransferase and related aminotransferases	Biosynthesis of glutamate
	COG0128	0.643	< 0.001	Amino acid transport and metabolism	5-Enolpyruvylshikimate-3-phosphate synthase	Biosynthesis of tryptophan|phenylalanine|tyrosine
	COG0548	0.684	< 0.001	Amino acid transport and metabolism	Acetylglutamate kinase	Biosynthesis of arginine
	COG0136	5.497	< 0.001	Amino acid transport and metabolism	Aspartate-semialdehyde dehydrogenase	Biosynthesis of lysine|methionine|leucine|isoleucine
	COG0129_1	5.193	0.009	Amino acid transport and metabolism	Dihydroxy acid dehydratase/phosphogluconate dehydratase	Biosynthesis of valine|leucine|isoleucine
	COG0405	0.901	< 0.001	Amino acid transport and metabolism	Gamma-glutamyltransferase	Biosynthesis of glutamate
	COG0069	6.373	< 0.001	Amino acid transport and metabolism	Glutamate synthase domain 2	Biosynthesis of glutamate
	COG0079	0.453	< 0.001	Amino acid transport and metabolism	Histidinol-phosphate/aromatic aminotransferase and cobyric acid decarboxylase	Biosynthesis of histidine
	COG0131	0.626	< 0.001	Amino acid transport and metabolism	Imidazoleglycerol-phosphate dehydratase	Biosynthesis of histidine
	COG0059_1	6.815	0.007	Amino acid transport and metabolism	Ketol-acid reductoisomerase	Biosynthesis of valine|leucine|isoleucine
	COG0520	0.459	0.007	Amino acid transport and metabolism	Selenocysteine lyase	Degradation of selenocysteine
	COG1171	0.820	< 0.001	Amino acid transport and metabolism	Threonine dehydratase	Degradation of threonine
	COG1063_1	0.459	< 0.001	Amino acid transport and metabolism	Threonine dehydrogenase and related Zn-dependent dehydrogenases	Degradation of threonine
	COG0633	7.877	< 0.001	Energy production and conversion	Ferredoxin	Photosynthesis/nitrogen fixation
	COG1145	0.872	< 0.001	Energy production and conversion	Ferredoxin	Photosynthesis/nitrogen fixation
	COG1146	− 1.512	< 0.001	Energy production and conversion	Ferredoxin	Photosynthesis/nitrogen fixation
	NOG05023	0.601	< 0.001	Energy production and conversion	PsaA and PsaB bind P700	Photosynthesis
	NOG05026	− 0.207	0.005	Energy production and conversion	Photosystem II D2 protein	Photosynthesis
	COG4242_1	1.144	< 0.001	Inorganic ion transport and metabolism	Cyanophycinase and related exopeptidases	Nitrogen fixation
	COG1348	0.418	0.040	Inorganic ion transport and metabolism	Nitrogenase subunit NifH	Nitrogen fixation
Down-regulated	COG1410	− 0.232	0.042	Amino acid transport and metabolism	Methionine synthase I, cobalamin-binding domain	Biosynthesis of methionine
	COG0111_2	− 1.642	< 0.001	Amino acid transport and metabolism	Phosphoglycerate dehydrogenase and related dehydrogenases	Biosynthesis of cysteine
	COG0498	− 0.446	< 0.001	Amino acid transport and metabolism	Threonine synthase	Biosynthesis of threonine
	COG0473_2	− 0.224	0.042	Energy production and conversion	Isocitrate/isopropylmalate dehydrogenase	Biosynthesis of leucine
	COG1613	− 2.184	< 0.001	Inorganic ion transport and metabolism	ABC-type sulfate transport system, periplasmic component	Sulfate uptake
	COG4208	− 0.296	0.002	Inorganic ion transport and metabolism	ABC-type sulfate transport system, permease component	Sulfate uptake
	COG0369	− 0.440	< 0.001	Inorganic ion transport and metabolism	Sulfite reductase, alpha subunit	Sulfur uptake

Only genes that had log2 fold changes with *p* < 0.05 are shown

A lower number of transcripts in the feather moss transcriptome was affected when co-incubated with the cyanobiont. This included a lower number of transcripts related to amino acid and sugar metabolism, with the up-regulation of a few genes related to synthesis of ATP and cellulose degradation, as well as the photosystem II reaction center protein M and an aminotransferase (Supporting Information Table S2, Supplementary Table 2). On the other hand, this transcriptome also presented the down-regulation of some genes encoding lipid and sugar metabolism, together with genes involved with glycolysis and electron transfer (Table 2; Supporting Information Table S2).

**Table 2:** Differential expression of genes involved with nutrient metabolism in the feather moss *H. splendens* after a week of co-incubation with the cyanobacterial strain UCTE CB012

Gene code		Log2 fold change	*p*	Metabolism	Function	Possible role in symbiosis
Up-regulated	COG0436	0.623	0.003	Amino acid transport and metabolism	Aspartate/tyrosine/aromatic aminotransferase	Metabolism of methionine|tyrosine|phenylalanine|tryptophan|novobiocin|alkaloids
	COG1472	0.991	0.032	Carbohydrate transport and metabolism	Beta-glucosidase-related glycosidases	Cellulose degradation
	COG0636	1.042	< 0.001	Energy production and conversion	F0F1-type ATP synthase, subunit c/archaeal/vacuolar-type H^+^-ATPase, subunit K	Synthesis of ATP
	NOG315236	1.073	< 0.001	Function unknown	Photosystem II reaction center protein M	Photosynthesis
Down-regulated	COG0696	− 0.884	0.003	Carbohydrate transport and metabolism	Phosphoglyceromutase	Glycolysis
	COG0438	− 0.794	0.014	Cell wall/membrane/envelope biogenesis	Glycosyltransferase	Synthesis of oligosaccharides and polysaccharides
	COG0221	− 1.403	0.014	Energy production and conversion	Inorganic pyrophosphatase	Metabolism of lipids
	COG5274_1	− 1.352	< 0.001	Energy production and conversion	Cytochrome b involved in lipid metabolism	Metabolism of lipids
	COG1290	− 0.629	< 0.001	Energy production and conversion	Cytochrome b subunit of the bc complex	Electron transfer
	COG0723	− 0.615	0.014	Energy production and conversion	Rieske Fe-S protein	Electron transfer

Only genes that had log2 fold changes with *p* < 0.05 are shown

## Discussion

We have observed that, after 1 week, gene expression in a cyanobacterium is deeply affected by contact with its host, the feather moss *H. splendens* (Figs. 1, 2, and 3a), especially in regards to energy production and conversion and amino acid metabolism (Fig. 4a). Vascular plants were previously shown to influence cyanobacterial transcriptomes, which present significant differences throughout the life cycles of their hosts [36]. However, little information is currently available for how cyanobacterial symbioses with bryophytes, characterized by very distinct developmental stages compared to vascular plants, are affected on a molecular level. This work thus suggests that cyanobacterial transcriptomes are also significantly influenced by contact with *H. splendens* gametophytes.

In contrast with the large number of genes that were differentially expressed in the cyanobacterial transcriptome, the results showed that this association has a much lower impact on the gene expression of *H. splendens* (Figs. 1, 2, and 3b). The expression of genes involved in defense and stress responses is often triggered in vascular plants following their contact with bacteria, cyanobacteria included [37]. Significant changes in the expression of these genes were not observed in this work, though (Supplementary Table 2). This also contrasts with a previous study that observed the down-regulation of host defense genes in the cyanobacteria–peat moss symbiosis triggered by physical contact [16]. The disparities between our work and previous works could be related to differences in some factors, including (a) hosts (*H. splendens* vs. *S. angustifolium*); (b) habitats (epiphytic on *Hylocomium* leaf surfaces vs. potentially endophytic inside *Sphagnum* hyalocytes); (c) co-incubation times (1 week vs. 3 to 4 weeks); or (d) cyanobiont (a *Nostoc*-like strain isolated from feather moss vs. a previously isolated strain from an undisclosed environment). On the other hand, ATP synthesis and cellulose degradation were up-regulated in *H. splendens* when co-incubated with the cyanobacterium (Table 2), suggesting modifications in the cell wall structure or cell expansion in the host.

It is worth highlighting, however, the significant number of genes with unknown functions that were differentially expressed by the host (Fig. 4a). Partially supporting our first hypothesis, N_2_ fixation was indeed up-regulated in the cyanobiont when in contact with its host (Table 1; Fig. 4b), which was likely a reaction to unknown bioactive metabolites released by *H. splendens* leading to an increase in cyanobacterial N_2_ fixation rates [19]. It is therefore possible that feather moss genes with currently uncharacterized functions have a role in influencing cyanobacterial activity in this interaction. Since little is known about the molecular and chemical ecology of moss–cyanobacteria symbioses, other uncharacterized genes are also likely to participate in these interactions to some unknown extent. Nevertheless, differential expression in genes related to cell differentiation in the co-incubated cyanobacterial genome was not observed, suggesting that any potential morphophysiological changes responsible for the increase in N_2_ fixation rates likely happened before the 1-week time point. On the other hand, we did not observe an up-regulation of N uptake in *H. splendens* under the co-incubated state, partially contradicting our first hypothesis. This may either suggest that the feather moss does not actually need N at this point of the symbiosis or that it requires no additional effort to take up N from its symbionts and the host can rely on constitutive mechanisms.

We found no transcriptomic evidence for a flow of S from the feather moss to the cyanobiont, refuting our second hypothesis. Changes in the expression of genes related to amino acid metabolism suggested that sulfur-containing amino acids are highly unfavored by the cyanobiont when in contact with *H. splendens*. In contrast with previous studies [16, 18], this suggests that, under the investigated conditions, there are insufficient sources of S, forcing the cyanobacterium to rearrange its amino acid profile and deflect resources for N_2_ fixation. S is an essential element in N_2_ fixation, either integrating the structure of the nitrogenase cofactors or as an essential part of metal-sulfur compounds that participate in other steps of the process [38]. Additionally, S is a core component of ferredoxin-like proteins, electron carriers that perform fundamental roles in processes like photosynthesis or N_2_ fixation [39, 40], for which two genes were significantly up-regulated in the cyanobiont. It is possible that more than 1 week is necessary for the host to provide nutrients to its cyanobiont, despite benefiting earlier from fixed N. It is also possible that distinct moss species affect cyanobacteria differently, as they have different requirements and growth strategies, especially when peat mosses and feather mosses are compared [41].

Contrarily to our third and fourth hypotheses, with the exception of a few genes, there was little to no evidence in our differential gene expression analyses for a significant down-regulation of photosynthetic genes in the cyanobiont and their up-regulation in the host after a week of physical contact. As N_2_ fixation is a very cost-intensive process, some sort of compensation for the increase in the diazotrophic activity presented by the cyanobacterium (Fig. 4b) would be expected. In other plant–cyanobacteria symbioses, this comes primarily in the form of carbon from the host [12]. It would be possible that the additional N fixed by the cyanobacterium was used for the growth of its own cells, but that would likely be followed by an equivalent increase in photosynthesis-related genes, which, with the exception of a single gene, was not observed (Table 1). Nevertheless, the maintenance of photosynthetic activity in the cyanobiont was also previously observed in symbioses with *P. schreberi* [18] and *S. angustifolium* [16]. This could indicate that, unlike more solid relationships with plants, it may not be a feasible strategy for cyanobacteria to rely on their hosts for carbon in epiphytic symbioses.

The nature of ecological relationships between organisms that live together is not static. Symbioses exist within a highly plastic spectrum composed of beneficial, neutral, or detrimental relationships with blurred borders [42]. Depending on biotic and abiotic conditions, the character of such ecological interactions can therefore oscillate in a continuum that may include mutualism, commensalism, neutralism, amensalism, and parasitism. Since mosses have not followed other bryophytes such as hornworts and some liverwort genera in evolving special structures to house cyanobionts [11], their interactions with cyanobacteria are likely to fluctuate more widely than those establishing stronger symbioses with these microbes.

## Conclusions

Although we did not observe noticeable changes in the host that would indicate an allocation of resources towards its cyanobiont, we did verify an undeniable increase in N_2_ fixation activity by the cyanobiont when in close contact with the moss. This suggests that, at least at the point when we evaluated this symbiosis, the nutrient flow might be going in a single direction, i.e., from the cyanobacterium to the host. Thus, if a mutual flow of nutrients also happens between *H. splendens* and cyanobacteria as suggested by research on other mosses, it does not seem to be a constant occurrence in these symbioses. On the other hand, it is also possible that nutrient exchange between mosses and cyanobacteria is not entirely based on active processes happening on demand, but rather by a passive leakage of constitutive nutrients from the host occurring regardless of contact with cyanobacteria or other external stimuli. Besides a lack of carbon from the host, the increase in N_2_ fixation without extra sources of S, nevertheless, leads the cyanobiont to change its amino acid profile against S-containing amino acids, likely to rearrange resources and be able to synthesize the molecules necessary for supporting the increase in N_2_ fixation promoted by the symbiosis. Therefore, epiphytic cyanobacterial symbioses could be more likely to fluctuate between mutualism and commensalism than those in which they are retained as endophytes.

### Supplementary Information

Below is the link to the electronic supplementary material.

Supplementary file1 (XLSX 26 KB)

Supplementary file2 (XLSX 9 KB)

## Data Availability

The RNAseq data obtained in this study was deposited in the NCBI GEO database under the accession numbers GSM7688832–GSM7688843.
